# Determinants of Cyanobacteria and Algae Diversity in Natural Freshwater Micro‐Ecosystems

**DOI:** 10.1111/1462-2920.70157

**Published:** 2025-07-29

**Authors:** Yasmin Rodrigues de Souza, Beatriz Melissa Campos, Fernando Miranda Lansac‐toha, Annika Busse, Jana S. Petermann, Gustavo Quevedo Romero, Pablo Augusto Poleto Antiqueira, Luzia Cleide Rodrigues

**Affiliations:** ^1^ Graduate Program in Ecology of Inland Water Ecosystems (PEA), Centre of Research in Limnology, Ichthyology and Aquaculture (Nupélia) State University of Maringá (UEM) Maringá Paraná Brazil; ^2^ Graduate Program of Compared Biology (PGB), Centre of Research in Limnology, Ichthyology and Aquaculture (Nupélia) State University of Maringá (UEM) Maringá Paraná Brazil; ^3^ Department of Environment and Biodiversity University of Salzburg Salzburg Austria; ^4^ Laboratório de Interações Multitróficas e Biodiversidade, Instituto de Biologia (IB) Universidade Estadual de Campinas (UNICAMP) Campinas Brazil; ^5^ School of Life Sciences University of Essex Colchester UK

**Keywords:** brightness, canopy cover, diversity patterns, phytotelma

## Abstract

In this context, estimating the contributions of single sites to overall beta diversity (LCBD—Local Contribution to Beta Diversity, i.e., indicator of site's ecological uniqueness) or partitioning overall beta diversity into contributions of individual species (SCBD—Species Contribution to Beta Diversity, i.e., degree of variation of individual species across the study area) has proven to be a good approach to improve the knowledge of drivers of beta diversity. The number of studies on beta diversity in hyperdiverse environments, such as the Neotropics, is still scarce. We explored the contributions of each site and species to the overall cyanobacteria and algae beta diversity of 77 natural freshwater micro‐ecosystems (i.e., tank bromeliads) of a neotropical ecosystem. We observed that LCBD was negatively related to Shannon diversity, turbidity and luminosity (% canopy cover). The negative relationship between LCBD and Shannon diversity indicates that micro ecosystems with less diversity reflect unique characteristics, and LCBD values can predict these environments. In our study, high LCBD values indicated environments in need of restoration, that is, poor in species richness and with greater turbidity and luminosity, showing that most bromeliad tanks presented high species diversity and low turbidity and luminosity.

## Graphical abstract

The Shannon Index, turbidity and luminosity negatively affected the LCBD, species with intermediate frequency of occurrence affected the LCBD, cyanobacteria and green algae are the groups that contribute most to the SCBD, as well as species with accessory frequency. LCBD can indicate microecosystems with greater need for management and conservation.

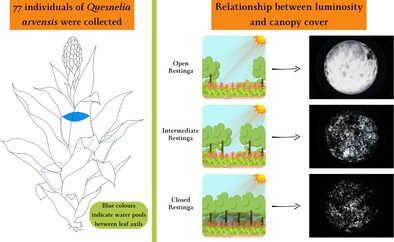

## 1 Introduction

Maintaining biodiversity is one of the greatest challenges today. Therefore, expanding our understanding of species distributions and the factors that influence community structure is critically important (Socolar et al. 2016). While it is common to evaluate how local factors affect ecological communities at small scales—typically through measures of alpha diversity such as species richness and abundance (Magurran 2013)—it is equally important to consider broader spatial scales. Regional diversity (gamma diversity), which encompasses the total number of species within a given region, and beta diversity, which reflects the variation in species composition among sites, also play crucial roles in biodiversity assessments (Magurran 2005; Anderson et al. 2011).

An important concept related to beta diversity is species frequency, defined as the proportion of sites in which a species occurs. This metric is fundamental for understanding patterns of species turnover and community similarity (Legendre and De Cáceres 2013). Species that occur frequently across sites may contribute to community homogenisation, thereby reducing beta diversity. In contrast, species with low frequency of occurrence can enhance dissimilarity among communities, increasing beta diversity. Importantly, beta diversity is closely linked to gradients in alpha diversity, and both are shaped by environmental filters such as habitat size, temperature, light availability and limitations in species dispersal across the landscape (Soininen et al. 2018).

Legendre and De Cáceres (2013) developed a method to estimate the contribution of each site (LCBD—Local Contributions to Beta Diversity) and each species (SCBD—Species Contributions to Beta Diversity) to overall beta diversity. Higher LCBD values at a given location may indicate environments with unique ecological conditions, often hosting distinct species assemblages (Silva et al. 2018). This approach aligns with concepts from niche theory, which proposes that species with narrow or marginal niches are typically more specialised, have more restricted geographic distributions, and are less locally abundant compared to species with broader, more generalist niches (Heino et al. 2005). As a result, generalist species are expected to occupy wider geographic ranges. The SCBD metric (Legendre and De Cáceres 2013) is closely related to this idea, as species with high SCBD values tend to be those whose presence significantly increases compositional differences among sites. Such species are often associated with more specific or marginal environmental conditions (Heino and Grönroos 2017; Silva et al. 2018).

Therefore, using LCBD and SCBD metrics to analyse community composition can reveal important theoretical patterns underlying beta diversity and offer valuable tools for conservation planning and biomonitoring. Within this framework, it is especially important to consider whether the uniqueness of a site's species composition (as captured by LCBD) is positively or negatively related to its alpha diversity (Landeiro et al. 2018).

In this context, many studies have found that high LCBD values occur in environments with low species richness (Holyoak et al. 2005; Chase et al. 2011; Heino and Grönroos 2017; Silva et al. 2018; Pozzobom et al. 2020; Quirino et al. 2021; Louchart et al. 2024). This information is relevant, as it allows conservation and management to prioritise certain actions, whether to preserve highly diverse environments or to recover a degraded area (Legendre and De Cáceres 2013). However, species richness is only one of the diversity metrics. The diversity measures resulting from indices (e.g., Shannon index, Equitability) can serve as indicators of the balance of ecological systems, functioning as a tool for environmental management (Magurran 2013).

Understanding how communities respond to biotic and abiotic variations is essential for developing effective biodiversity conservation strategies. Biodiversity is a dynamic and ever‐evolving phenomenon, shaped by ecological interactions and environmental changes. By uncovering the mechanisms that drive community composition and organisation, we can improve our ability to predict ecosystem responses to disturbances and design targeted conservation efforts that safeguard ecological integrity (Heywood 1995). Previous studies showed that environmental characteristics lead to changes in the species composition, affecting each form of life differently and, consequently, the degree of species occupation and patterns of LCBD (Schneider et al. 2015). The increase in habitat size, for example, has been shown to alter species composition and cause an increase in richness in different biological groups (Srivastava 2006; Haddad et al. 2015; Padisák et al. 2016; Busse et al. 2018). This is because, according to species‐area relationship theory (MacArthur and Wilson 1967; Connor and McCoy 1979), the largest habitats support the largest number of species (MacArthur and Wilson 1967). The greater diversity and abundance of large habitats can also be explained by a greater niche availability (Dodson 1991; Antiqueira, Petchey, dos Santos, et al. 2018).

Other abiotic factors are also important for structuring communities, such as light availability (Reynolds 2006; Loverde‐Oliveira et al. 2012) and structural complexity (Tews et al. 2004). Light availability is a determining factor in structuring the community of primary producers (Margalef 1978; Reynolds 1998; Bortolini, Train, and Rodrigues 2016). Light exposure also influences thermal variations, where environments more exposed to light tend to have a greater diurnal variation in their temperature than environments that are less exposed (Busse et al. 2018), and these variations can directly affect the local species richness (Kratina et al. 2017).

Primary producers such as cyanobacteria and algae play a central role in aquatic ecosystems and are highly sensitive to environmental fluctuations. They drive primary productivity, contribute to major biogeochemical cycles and respond rapidly to changing conditions (Reynolds 1988; Bortolini, Moresco, et al. 2016). Their capacity, or lack thereof, to adapt to environmental variability, particularly shifts in light and temperature, is closely linked to their morphological diversity, as they include species with a wide range of sizes and forms (Lewis 1976; Margalef 1978). Consequently, both biotic and abiotic factors, such as resource availability (e.g., light, oxygen, temperature, turbidity and nutrients), hydrodynamic conditions (e.g., water flow and column stratification) and herbivory, can significantly influence their morphology, species richness and abundance (Morabito et al. 2007; Naselli‐Flores and Barone 2011).

Investigating the structure of biological communities in natural systems is not an easy task, especially considering the difficulty of replication. The use of micro‐ecosystems is an excellent tool that overcomes these difficulties (Srivastava et al. 2004). Natural micro‐ecosystems, such as those found in tank bromeliads, have been widely used as model systems to test ecological theories (Kitching 2000; Srivastava 2006; Srivastava et al. 2008; Romero and Srivastava 2010; Starzomski et al. 2010; Farjalla et al. 2016) as they are easy to sample and can be measured in their entirety (Srivastava et al. 2004). Represented by more than 2600 species, bromeliads are considered the largest exclusively neotropical family of flowering plants, are widespread in tropical climates, and are well‐known phytotelmata plants (Balke et al. 2008). The leaf anatomy of tank bromeliads allows water tanks to form between the axils of the leaves, storing rainwater as well as leaf litter from the canopy (Srivastava 2006). These tanks are conducive to the development of many groups of organisms, which are transported by wind or through animal vectors (Buosi et al. 2014) such as protists, small metazoans, insect larvae, algae and cyanobacteria (Busse et al. 2018; Antiqueira et al. 2022). Therefore, these plants play a role as shelters for different biological groups such as primary producers, consumers and decomposers. Among the environmental factors shaping bromeliad tanks is canopy cover due to its direct connection with light availability. Canopy cover is affected by anthropogenic factors such as deforestation. The absence or partial canopy cover facilitates the entry of light into the phytotelmata (Guimaraes‐Souza et al. 2006; Poniewozik et al. 2020). Thus, lower canopy cover rates in open regions of Restinga, a type of Atlantic rainforest on coastal dunes, favour the development of organisms in bromeliads (Guimaraes‐Souza et al. 2006; Poniewozik et al. 2020). On the other hand, high amounts of allochthonous material and its high decomposition rates cause an increase in water turbidity, which can prevent the entry of light and, consequently, be a limiting factor for many species of algae (Farjalla et al. 2016). Therefore, as canopy cover and water turbidity are variables of great importance for the development of this ecosystem (Hegner 1926; Addicott 1974), they may affect the diversity patterns of aquatic organisms such as primary producers. Consequently, these variables are expected to have a strong influence on LCBD patterns. Ramos and Moura (2019) presents a review of the ecological importance of algae and cyanobacteria, noting that they account for a significant portion of the total microbial biomass found in tanks. These organisms contribute in multiple ways to the ecological dynamics of these environments, serving as the foundation of the community trophic chain, acting as major sources of carbon and participating in nutrient cycling processes. The nutrients can be absorbed both by the trichomes of bromeliads and by associated organisms (Laessle 1961; Bermudes and Benzing 1991; Brouard et al. 2011).

Thus, the objective of this work was to investigate the influence of taxonomical diversity components (species richness, Shannon and Equitability), habitat size (tank‐bromeliad volume), light incidence (canopy cover) and other abiotic variables (e.g., dissolved oxygen, temperature and turbidity) on the local (LCBD) and species (SCBD) contributions for the beta diversity of cyanobacteria and algae in 77 natural micro‐ecosystems. First, we hypothesise that LCBD will be negatively related to cyanobacteria and algal diversity (H1). This expectation is based on the premise that higher diversity reduces the uniqueness of individual bromeliad communities compared to others. Second, as a consequence of H1, we hypothesise that environmental variables known to promote algal diversity—higher light availability, larger volume and higher temperature—will be negatively associated with LCBD, whereas turbidity will show a positive relationship (H2). These predictions are supported by previous findings indicating that such environmental conditions favour algal richness and abundance (Reynolds 2006). Third, given that SCBD values are derived from total community variance and that species with intermediate frequency (i.e., accessory species) typically contribute more to beta diversity than widely distributed (i.e., common) species (Heino and Grönroos 2017), we hypothesise that SCBD will exhibit a quadratic relationship with species frequency, with accessory species making the highest contributions to community variation (H3). Finally, we hypothesise that different taxonomic groups will contribute differently to beta diversity due to their distinct ecological and environmental responses (H4). This variation may reflect differences in dispersal ability, tolerance to environmental conditions and functional traits among cyanobacteria and algae.

## 2 Methodology

### 2.1 Study Location and System

The study was conducted at Ilha do Cardoso State Park, municipality of Cananéia, on the southern coast of São Paulo State, Brazil (25°03′ S, 48°53′ W), in September 2013, at the beginning of the rainy season. Ilha do Cardoso is characterised by average annual temperatures between 20°C and 22°C and average annual rainfall of 2250 mm (Pessenda et al. 2012), and the relative humidity is higher than 66% in spring (sampling season of the present study) (Manoel and Mota 2012). The study was carried out in the northern part of the island, in an area of 4.5 km in length. The site is situated in the Restinga rainforest, (i.e., an Atlantic rainforest on the coastal dunes) (Rizzini 1997). The Restinga rainforest on Ilha do Cardoso shows different vegetation and abiotic conditions along a gradient of canopy cover. This gradient ranges from a less forested Restinga (i.e., more sun‐exposed habitats for bromeliads and their microbiota) to a more forested Restinga environment (i.e., shady habitats for bromeliads and their microbiota). In the less wooded habitat, shrubby vegetation (maximum 4 m tall) is distributed in areas containing lianas with areas exposed to the sun between these areas. In the more wooded habitat, trees range from 6 to 8 m in height and can form a relatively continuous canopy cover. The density of bromeliads was higher in the more forested Restinga (Busse et al. 2018).

**FIGURE 1:**
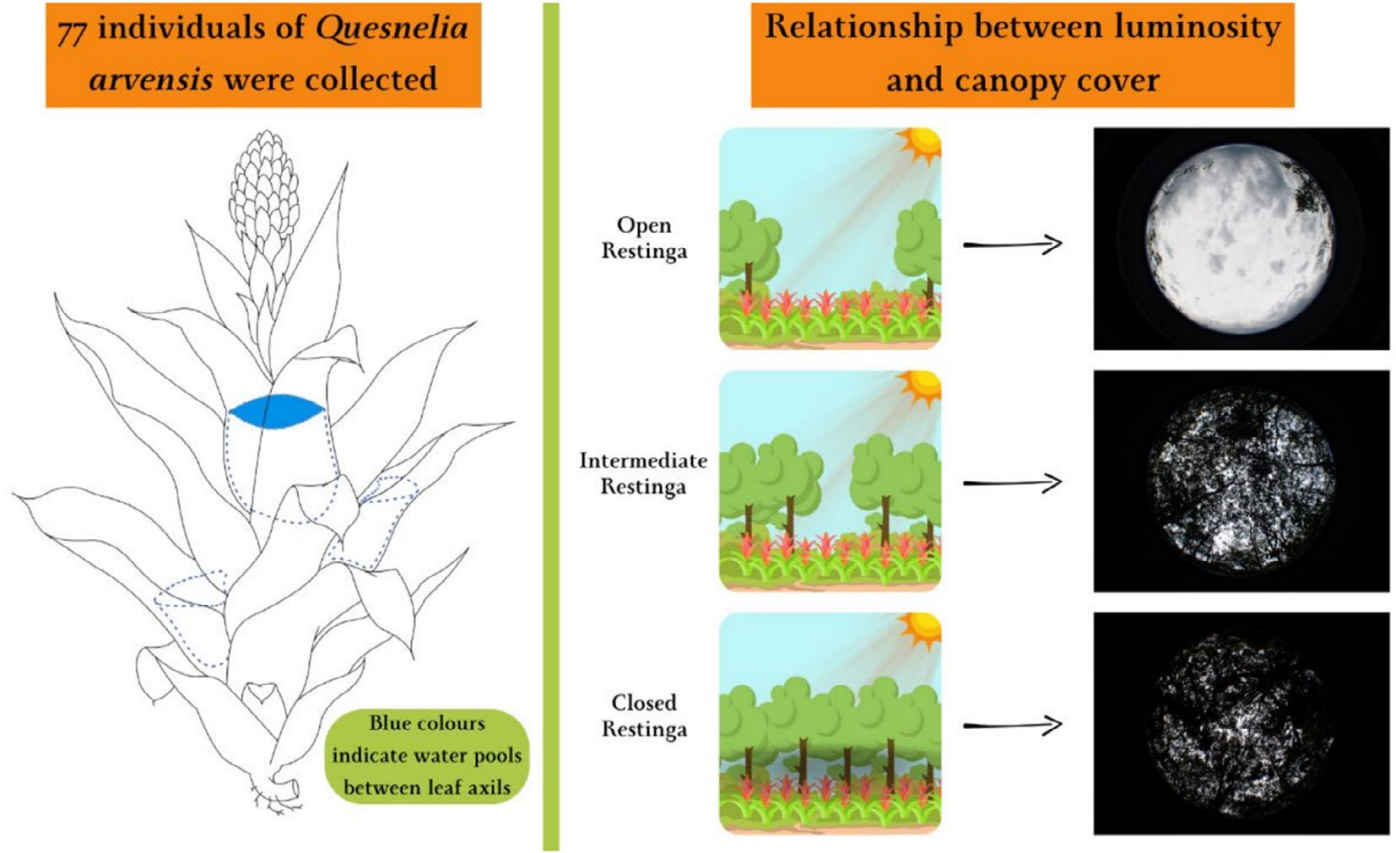
Schematic drawing of the tank bromeliad of the species *Quesnelia arvensis* Mez. (Bromeliaceae) used in this study and the canopy cover gradient. *Source:* Prepared by the author.

Tank bromeliads occur almost exclusively in the Neotropics and the species used in this study, *Quesnelia arvensis* (Vell.) Mez. (Figure 1), is exclusively neotropical and can grow at ground level or as epiphytes on trunks or branches. Its funnel‐shaped leaf morphology, with numerous leaf compartments, facilitates the capture of rainwater and litter that falls from the canopy. The tanks formed in the leaf axils accumulate rainwater and make these plants micro freshwater ecosystems. The water held by plants is commonly known as phytotelmata, of which bromeliads constitute only one possible type (Kitching 2000).

## 3 Sampling

### 3.1 Cyanobacteria and Algae Communities

The cyanobacteria and algae communities were sampled using pipettes from the central tanks of *Q. arvensis* bromeliads (Figure 1). Sampling was conducted across three areas with varying canopy coverage, representing different levels of sunlight exposure: open, forested and intermediate areas (hereafter referred to as open, closed and intermediate Restinga). A total of 77 ground‐level tank bromeliads were randomly selected, with each considered a distinct microcosm. To ensure comparability, we randomly selected bromeliads of similar size based on their total volume, maintaining a minimum distance of 10 m between selected bromeliads. The central tank water volume for each bromeliad was sampled and measured (mean ± SE: 9.44 ± 7.43 mL), with actual measured values used in the analyses. For better visualisation of the data (Supporting Information), the volumes were categorised as small (0.5–4.5 mL), medium (5.1–11.1 mL) and large (11.5–30 mL). Sampling was carried out under licence 23,689^−1^, issued by the Chico Mendes Institute for Biodiversity Conservation (ICMBio).

The samples for the study of cyanobacteria and algae were fixed with Lugol acetic solution (5%). The identification of taxa was carried out using specialised literature: (Bicudo and Bicudo 1970; Komárek and Fott 1983; Krammer and Lange‐Bertalot 1986, 1988, 1991). The counting of individuals (cells, coenobia, colonies, or filaments) was performed according to the method of Utermöhl (1958) and Lund et al. (1958) after previous sedimentation of the sample using the inverted microscope at 400× magnification. Density was calculated according to (APHA 2005), and the result was expressed in individuals per millilitre (ind mL^−1^).

The recorded taxa were grouped into four groups of algae based on taxonomic and ecological characteristics. (1) *Phytoflagellates* (classes Chlamydophyceae, Cryptophyceae and Euglenophyceae): includes flagellate organisms that are potentially heterotrophic (Reynolds 2006) and that have been shown to be favoured in environments with high concentrations of organic matter (Bellinger and Sigee 2011). (2) *Diatoms* (classes Bacillariophyceae, Coscinodiscophyceae and Mediophyceae): includes organisms that have a silica shell and that are often favoured in environments with high mixed water column (Bellinger and Sigee 2011). (3) *Green algae* (classes Chlorophyceae, Oedogoniophyceae and Trebouxiophyceae): includes organisms that are favoured by environments with high light availability and intermediate water column mixing (Jensen et al. 1994; Reynolds 2006). (4) *Cyanobacteria* (Cyanobacteria division): composed of prokaryotic organisms (without a true cell nucleus) with the potential to produce toxins (Whitton and Potts 2002), which are favoured in environments of low mixing and high concentration of nutrients (Bellinger and Sigee 2011). These groups have demonstrated sensitivity to environmental variability and great utility for the biomonitoring of trophic and hydrodynamic conditions in freshwater aquatic systems. The frequency (C) of species was calculated according to the equation of Dajoz (1973), where the frequency values of the species were categorised as *constant* (C equal to or greater than 50%), *accessory* (C less than 50% and equal to or greater than 25%) and *rare* (C less than 25%).

Alpha diversity (α = species richness) was considered as the number of taxa present in each sample and gamma diversity (γ) as the total number of species in all samples. As a measure of diversity in each bromeliad, the Shannon‐Weaver Diversity Index (*H*′) was calculated (Shannon and Weaver 1963). Evenness (*E*), as a measure of how homogeneously the density is distributed among species, was also estimated according to Pielou (1966): *J* = *H*′/*H*_maximum_, where *H*′_maximum_ is the maximum possible diversity that can be observed if all species are present in equal abundance.

### 3.2 Environmental Variables in Bromeliads

Different biotic and abiotic parameters were measured in the study. The water temperature inside the bromeliad phytotelmata was measured using portable digital temperature readers (Thermochron iButton DS1921G), inserted in all bromeliads and recording the water temperature of each bromeliad for 23 h (before sampling). From these data, the mean temperature, maximum water temperature and coefficient of variation were calculated, which were strongly correlated (Pearson correlation: *p* ≤ 0.001 for all pairs of correlations) (Hirschmann et al. 2008). Thus, to avoid multicollinearity in the analyses, we chose a priori one of the three variables for further analysis. We chose the coefficient of variation because it better represents the variability.

Canopy coverage (%) was determined for each bromeliad by analysing canopy photos taken above the centre of each bromeliad, using a camera with a 35 mm lens. The photos were analysed using ImageJ 1.48v software (http://imagej.nih.gov/ij/). The dissolved oxygen concentration (%) and pH were measured in each bromeliad using a portable multiparameter meter (cyberscan PD 650, Oaklon). A water sample was collected to analyse turbidity (NTU) and ammonium concentration (μM) using a portable fluorometer (AquaFluor).

### 3.3 Data Analysis

All analyses were performed in the R program (R Development Core Team 2025). The plots were made using the ggplot2 package (Wickham 2011), while the function and package for each analytical step are described below. Pearson correlations were performed to verify the multicollinearity between the variables. In this case, ammonium, pH and turbidity were highly correlated (*r* > 0.7 in all comparisons) and the first two were excluded from the analyses.

First, to test the relationship between the structure of the algal community (i.e., richness, abundance, Shannon Index and Equitability) with the different abiotic components, linear regressions were performed including all predictor variables and community descriptors as response variables. For this, we used the lm function of the stats package (The R Stats Package). The same approach was carried out with the richness and abundance of each group (i.e., green algae, cyanobacteria, diatoms and phytoflagellates). To calculate the Shannon index and Equitability, the entire algal communities were used.

The local contribution to beta diversity (LCBD) and species contribution to beta diversity (SCBD) were obtained according to the procedures described by (Legendre and De Cáceres 2013), using the beta.div function of the adespacial package (Dray et al. 2018). For this analysis, the Hellinger transformation was applied over the complete matrix with data on the abundance of algae.

To evaluate the relationship between LCBD and diversity indexes (H1), as well as with abiotic variables (H2), beta regression models were performed using the betareg function of the betareg package (Cribari‐Neto and Zeileis 2010). Beta regression is appropriate in this context because it assumes that the dependent variable has a restricted continuous value between 0 and 1 (as is the case with the LCBD), in a way that reduces the heterogeneity and asymmetry of the data (Cribari‐Neto and Zeileis 2010). Environmental variables (except pH) were previously logarithmised.

To test H3, linear (or quadratic) beta regression models with the first and second order terms were performed between the SCBD (response variable) and the frequency of occurrence of the species (predictor variable). The second‐order term was used to allow the model curve to decrease after the peak, as expected. The Akaike Information Criterion (AIC) was used to determine which model best fits the data. Each group of algae was analysed separately.

Finally, to understand whether the groups of algae differ in SCBD (H3), an analysis of variance (ANOVA; aov function of the stats package) was performed, followed by the Tukey test (TukeyHSD) function of the stats package for assessing differences between groups.

## 4 Results

### 4.1 Algal Community

**FIGURE 2:**
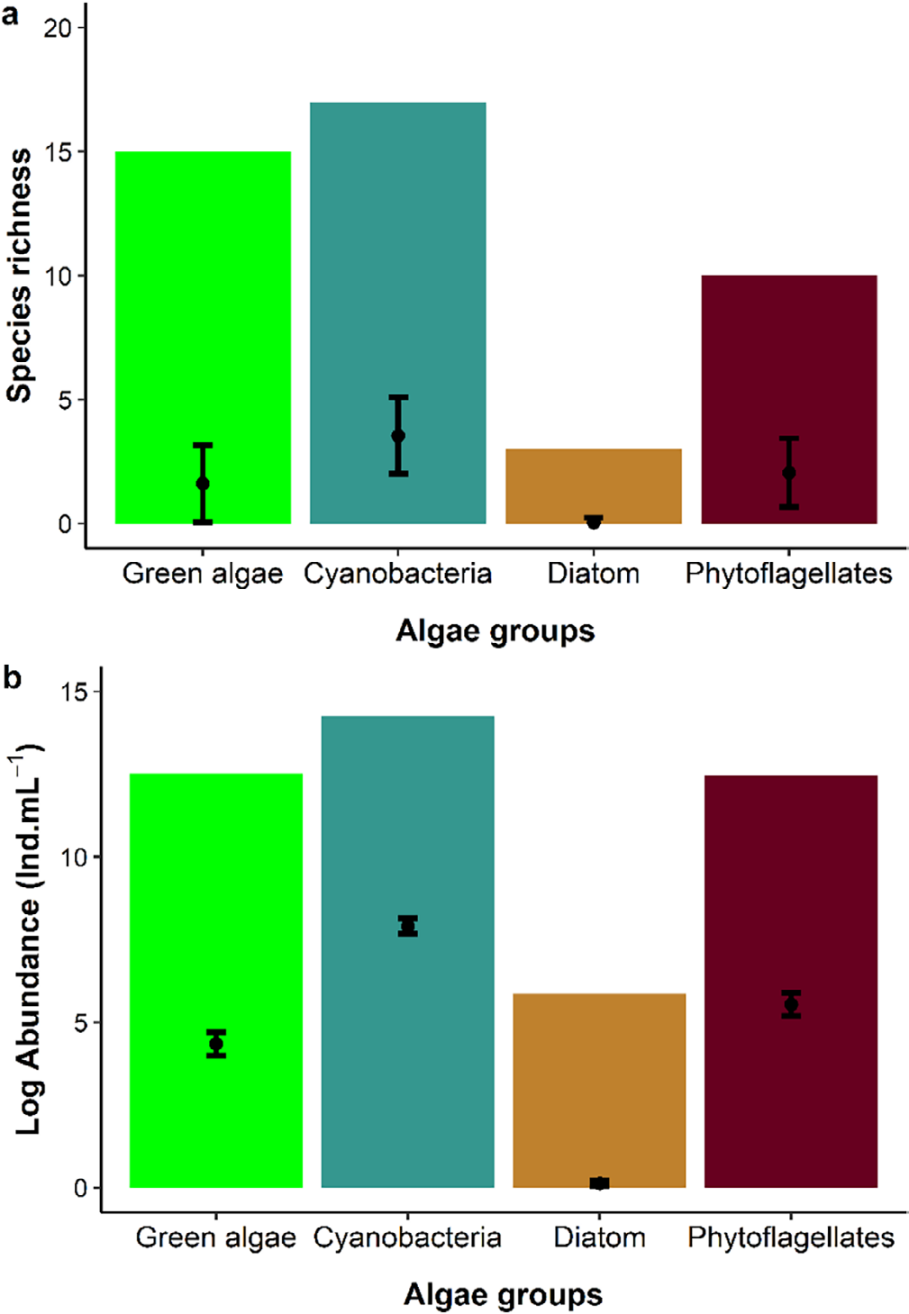
Species richness and abundance of each group of cyanobacteria and algae in bromeliad tanks in Brazilian Restinga forest. The bars represent calculated values for all sampling points, while the error graph (mean and standard error) corresponds to the calculation made for each micro‐ecosystem.

Forty‐five algal taxa were identified in the analysed micro‐ecosystems (γ diversity) (Supporting Information), ranging from 2 to 16 taxa per phytotelma and an average of 7 taxa (β diversity). Cyanobacteria had the highest number of taxa (16%–38% totally) and the highest average (4 taxa) per phytotelma (Figure 2a). Green algae were represented by 15 taxa (33%), most of them belonging to green algae, with an average of two taxa per environment (Figure 2a). Ten taxa (22%) of phytoflagellates were recorded, mainly represented by euglenophyceans. Only three diatom taxa (7%) were recorded, with an average richness of one taxon per plant (Figure 2a). The results of species frequency, considering the 77 plants sampled, showed a high contribution of rare species (73.33%), represented mainly by green algae with 13 taxa, followed by cyanobacteria with ten taxa, phytoflagellates with seven taxa and diatoms with three taxa. Accessory species contributed 15.56%, represented by seven taxa, except for diatoms. The species classified as common corresponded to 11.11%, distributed in five taxa, three cyanobacteria and two phytoflagellates (Supporting Information).

The density ranged from 46 to 420 ind mL^−1^, with a mean of 27 ind mL^−1^. Cyanobacteria had the highest mean values per microcosm, 20,392 ind mL^−1^ and a total of 1,570,153 ind mL^−1^ (Figure 2b). Green algae were the second group with the highest contribution, with 270,112 ind mL^−1^ recorded (or 12.5 in log scale) and an average per sample of 3368 ind mL^−1^ (4.3 in log). Phytoflagellates showed 259,338 ind mL^−1^ (12.4 in log), and an average higher than 3507 ind mL^−1^ (5.5 in log) (Figure 2b). Diatoms were less abundant (Figure 2b). The Shannon index ranged from 0 to 2.1, with a mean value of 1.1, and evenness ranged from 0.1 to 1, with a mean of 0.5 (Figure 4).

### 4.2 Differences in Environmental Variables Between Different Habitat Types

The percentage of canopy cover showed the lowest average value and high variability in the micro ecosystems collected in the open Restinga 37.5 (48.7) (mean values, coefficient of variation [in parentheses]) (Table 1). There were higher mean values of dissolved oxygen 38.4 (57.37), temperature 24.11 (15.81), pH 4.73 (12.15), turbidity 40.8 (47.99) and ammonium 512.34 (60.3) in the open Restinga micro‐ecosystems. Higher average volumes of the central tank were found in the intermediate and closed Restinga, with high variability (CV > 80) in all areas.

**TABLE 1:** Mean values, coefficient of variation (in parentheses), minimum and maximum values (bold) of environmental variables in bromeliad micro‐ecosystems, categorised by % canopy cover (open, intermediate and closed Restinga).

	Open	Intermediate	Closed
OD (%)	38.4 (57.37) **4.2–87.1**	28.98 (49.57) **6.9–59.3**	26.57 (34.69) **13–46.4**
Temperature (°C)	24.11 (15.81) **19.8–35.1**	22.31 (11.28) **20.1–29**	23.5 (14.78) **18.5–29**
pH	4.73 (12.15) **3.7–5.78**	4.38 (9.28) **3.75–5.35**	4.36 (7.14) **3.81–5.08**
Central tank volume (mL)	9.71 (54.06) **3–23**	11.75 (80.84) **1–30**	6.6 (89.53) **0.5–24**
Turbidity	40.8 (47.99) **9.86–77.22**	33.38 (46.67) **3.48–65.32**	22.17 (66.46) **4.63–64.19**
Ammonium	512.34 (60.3) **94.68–1373**	400.45 (93.13) **46.51–1379**	180.88 (127.96) **50.75–1234**
Canopy cover (%)	37.5 (48.7) **7.59–67.59**	75.36 (7.24) **63.66–85.07**	76.67 (6.7) **65.97–88.15**

### 4.3 Relationship of Diversity, Richness and Equitability With Environmental Variables

**FIGURE 3:**
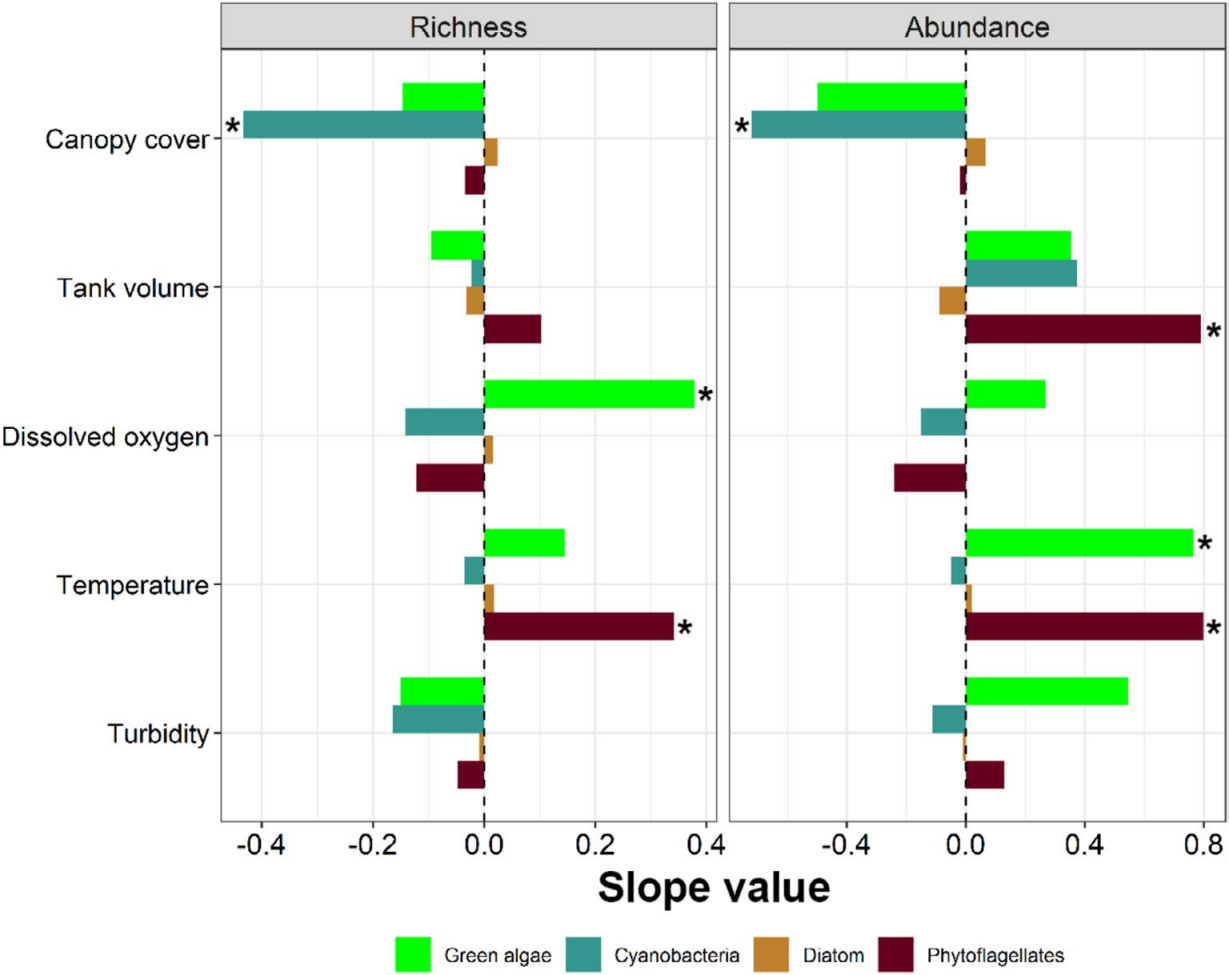
Relationship between species richness (left) and abundance (right) (response variables) of each group of cyanobacteria and algae in tank bromeliads with environmental variables (predictor variables). The bars represent the estimated coefficients of the regression (slope), where positive values mean a positive effect, and negative values mean a negative effect. Asterisk (*) indicates significant relationships (*p* < 0.05); only the significant variables were plotted.

Overall, only the canopy cover significantly impacted the whole algal community in the study, negatively influencing the abundance and positively influencing the Equitability (Table 2) of the algae. Analysing the species richness (number of taxa) of each group separately, the results showed that the different groups were influenced by effects of environmental variables (Figure 3). The species richness of cyanobacteria was negatively affected by canopy cover (*p* = 0.047, Figure 3), green algae were positively affected by dissolved oxygen (*p* = 0.041, Figure 3) and phytoflagellates were positively affected by temperature (*p* = 0.04, Figure 3).

**TABLE 2:** Relationship between total taxon richness, abundance, Shannon index and Equitability with environmental variables in tank bromeliads using linear or quadratic model.

Index	Effect	SE	Z	*p*
Richness (2.4%)				
(Intercept)	7.247	0.328	22.120	**< 0.001**
Canopy cover (%)	−0.590	0.396	−1.490	0.140
Tank volume (mL)	−0.047	0.341	−0.140	0.890
Dissolved oxygen (%)	0.131	0.372	0.350	0.730
Temperature (°C)	0.469	0.340	1.380	0.170
Turbidity (NTU)	−0.371	0.359	−1.030	0.310
Abundance (14.4%)				
(Intercept)	27,272	7126	3.830	**0.000**
Canopy cover (%)	−27,278	8601	−3.170	**0.002**
Tank volume (mL)	10,377	7405	1.400	0.165
Dissolved oxygen (%)	−3860	8096	−0.480	0.635
Temperature (°C)	8314	7393	1.120	0.265
Turbidity (NTU)	−1795	7811	−0.230	0.819
Index of Shannon (1.0%)				
(Intercept)	1.100	0.058	19.130	**< 0.001**
Canopy cover (%)	0.073	0.069	1.050	0.300
Tank volume (mL)	0.007	0.060	0.110	0.910
Dissolved oxygen (%)	−0.041	0.065	−0.630	0.530
Temperature (°C)	0.025	0.060	0.410	0.680
Turbidity (NTU)	0.053	0.063	0.840	0.410
Evenness (8.1%)				
(Intercept)	0.508	0.023	22.330	**< 0.001**
Canopy cover (%)	0.067	0.027	2.430	**0.017**
Tank volume (mL)	0.001	0.024	0.040	0.968
Dissolved oxygen (%)	−0.011	0.026	−0.410	0.682
Temperature (°C)	−0.022	0.024	−0.950	0.346
Turbidity (NTU)	0.045	0.025	1.810	0.074

*Note:* Percentage (%) indicates the explanation of the predictor variables on the diversity metrics based on the adjusted *R* ^2^ values of the model. Statistical significance (*p* < 0.05) indicated in bold.

Regarding abundance, cyanobacteria were negatively affected by canopy cover (*p* = 0.012, Figure 3). Phytoflagellate abundance was positively affected by temperature (*p* = 0.027, Figure 3) and tank volume (*p* = 0.029, Figure 3). Green algae were also positively influenced by temperature (*p* = 0.026, Figure 3).

### 4.4 The Local Contribution to Beta Diversity (LCBD)

**FIGURE 4:**
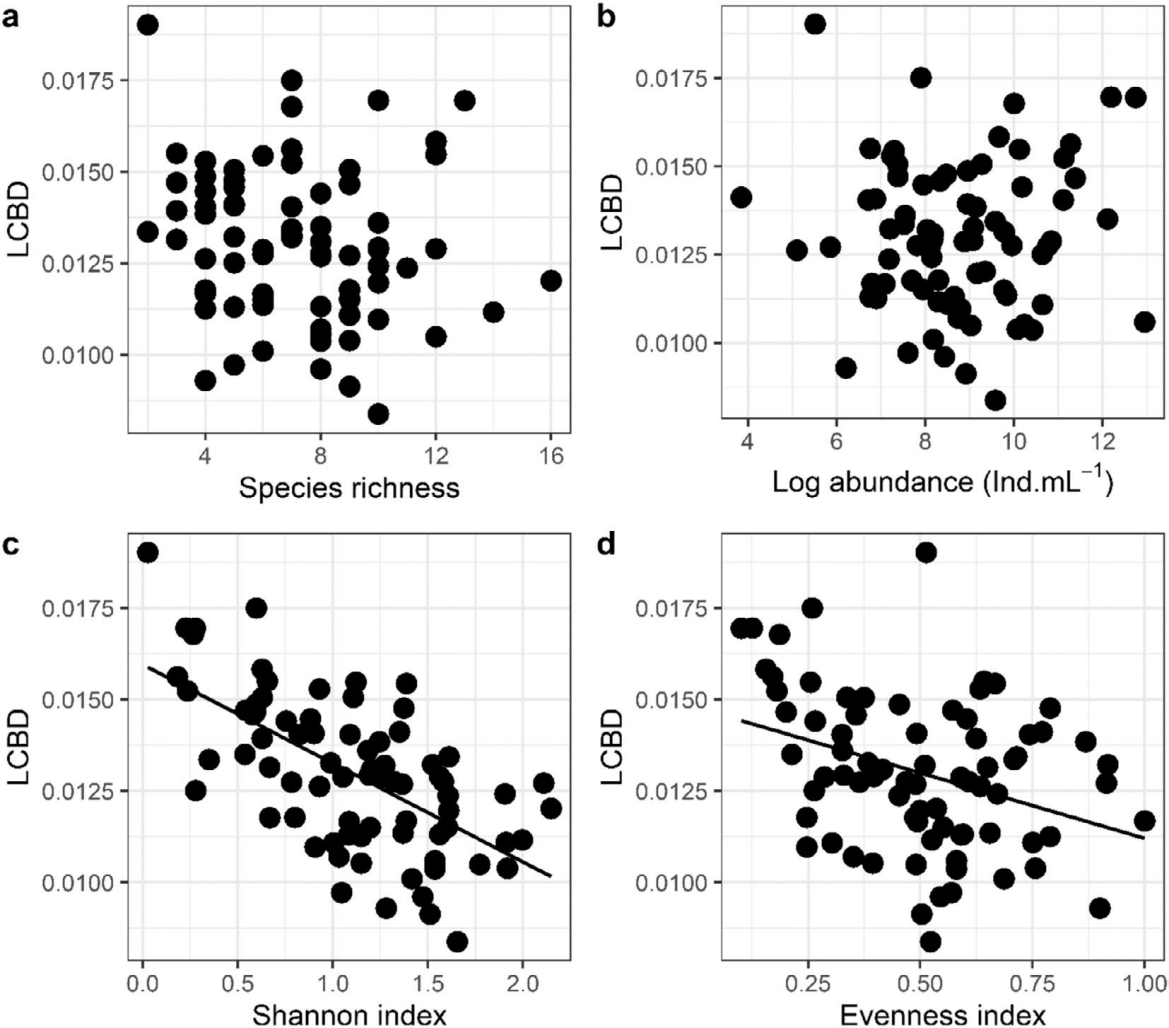
Relationship between LCDB and species richness (a), abundance (b), Shannon Index (c) and Equitability (d) in tank bromeliads. The regression line is shown only for variables that were significant using a linear model.

**FIGURE 5:**
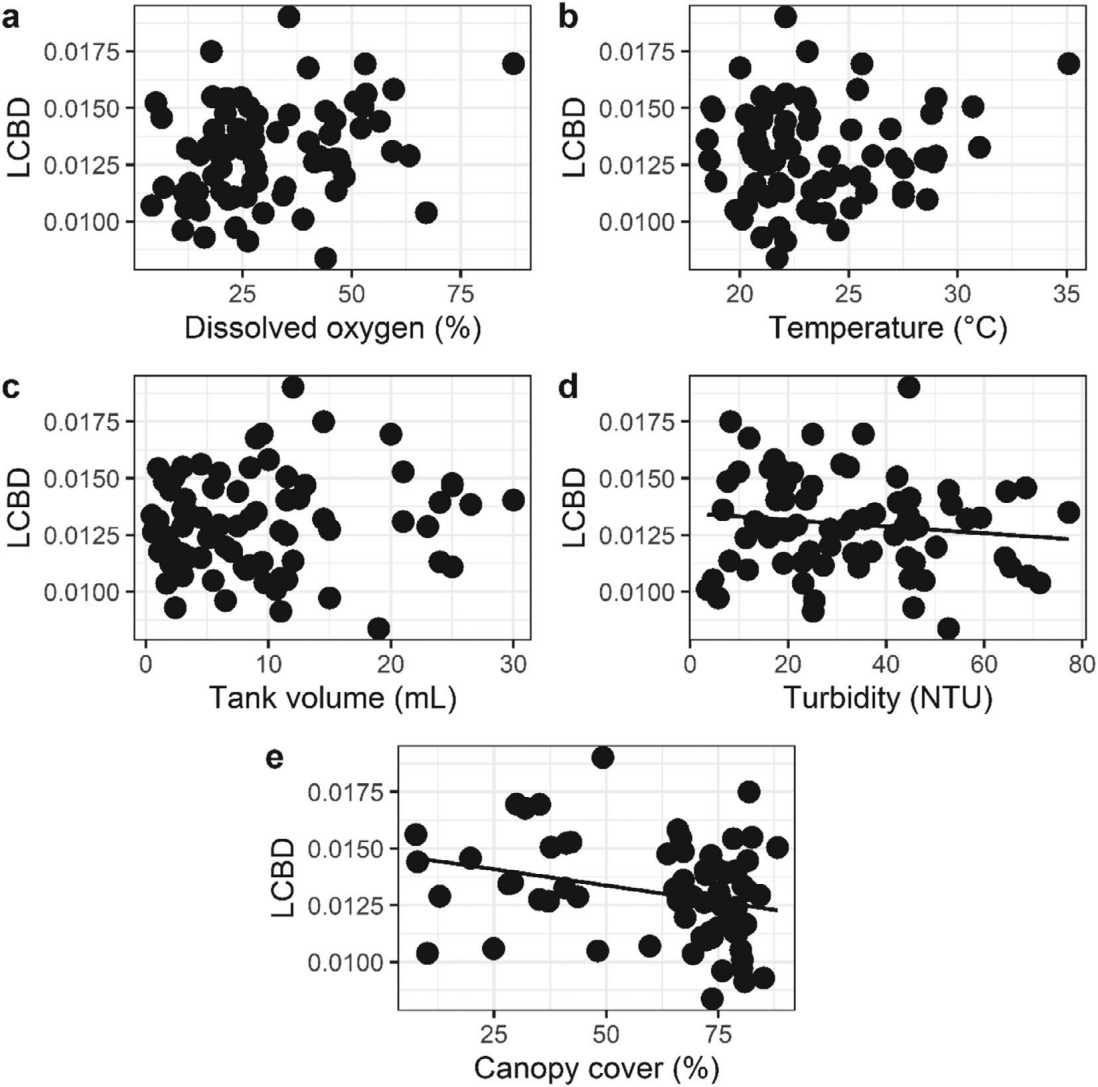
Relationship between LCDB and environmental variables. The regression line is shown only for variables that were significant.

The local contribution to beta diversity (LCBD) was negatively associated with the Shannon Index (*p* < 0.001) and evenness (*p* = 0.001) and was not affected by species richness (*p* = 0.105) or abundance (*p* = 0.240) (Table 3, Figure 4). Turbidity (*p* = 0.028) and canopy cover (*p* = 0.014) also negatively affected the LCBD. The other abiotic variables did not influence the variation patterns of the LCBD (Table 3; Figure 5).

**TABLE 3:** Relationship of the LCBD with the richness of total taxa, Shannon index, Equitability and environmental variables in tank bromeliads using the linear or quadratic model.

	Effect	SE	*Z*	*p*
(Intercept)	−4.256	0.049	−86.798	**< 0.001**
Richness of species (3.1%)	−0.010	0.006	−1.623	0.105
(Intercept)	−4.351	0.026	−169.630	**< 0.001**
Abundance (1.5%)	**0.001**	0.000	1.180	0.240
(Intercept)	−4.104	0.034	−119.908	**< 0.001**
Index of Shannon (37.4%)	−0.211	0.029	−7.159	**< 0.001**
(Intercept)	−4.191	0.045	−92.942	**< 0.001**
Evenness (11.4%)	−0.282	0.085	−3.329	**0.001**
Environmental variables (16.5%)				
(Intercept)	−4.188	0.166	−25.264	**< 0.001**
Dissolved oxygen (%)	0.002	0.001	1.401	0.161
Temperature (°C)	0.001	0.005	0.137	0.891
Tank volume (mL)	0.001	0.002	0.374	0.709
Turbidity (NTU)	−0.002	0.001	−2.195	**0.028**
Canopy cover (%)	−0.002	0.001	−2.467	**0.014**

*Note:* Percentage (%) indicates the explanation of the predictor variables on the diversity metrics based on the adjusted Pseudo‐*R* ^2^ values of the model. Statistical significance between LCDB values and measures of community diversity and abiotic variables are indicated in bold.

### 4.5 Contribution of Species to Beta Diversity (SCBD)

**FIGURE 6:**
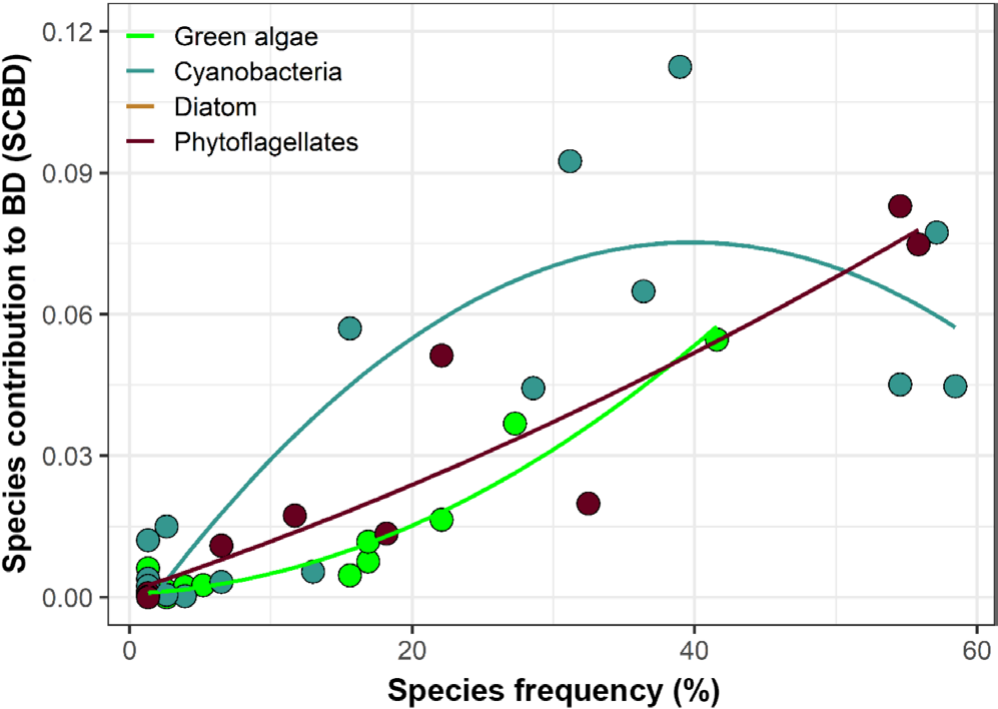
Relationship between species contribution to beta diversity (SCBD) and species frequency in large alga groups.

The relationship between SCBD and green algae was best described by a second‐order polynomial relationship (Table 4). The model showed very high predictive power, explaining approximately 93.4% of the SCBD variation. This group showed an increasing curvilinear relationship, where low values along the gradient of frequency of occurrence had little effect on SCBD, but from 20% it grew exponentially (Figure 6). This increase was mainly caused by *Oocystis* sp. (41.6%) and *Oedogonium reinschii* J. Roy ex Hirn (27.7%).

**TABLE 4:** Relationship between occupancy of the sites in the sample (frequency of occurrence of algal and cyanobacterial groups) and species contribution values for beta diversity in tank bromeliads, based on linear or quadratic models.

Groups	Effect	SE	*Z*	*p*	Pseudo‐*R* ^2^ adj.	AIC
Green algae						
(Intercept)	0.010	0.001	9.360	**0.000**		
Frequency^a^	0.055	0.004	13.600	**0.000**	0.851	−106.705
Frequency^b^	0.017	0.004	4.180	**0.001**	0.934	−118.188
Cyanobacteria						
(Intercept)	0.034	0.005	7.310	**0.000**		
Frequency^a^	0.108	0.019	5.620	**0.000**	0.524	−73.1564
Frequency^b^	−0.065	0.019	−3.380	**0.004**	0.719	−81.2999
Phytoflagellates						
(Intercept)	0.027	0.004	6.540	**0.000**		
Frequency^a^	0.086	0.013	6.570	**0.000**	0.839	−55.4846
Frequency^b^	0.006	0.013	0.487	0.641	0.822	−53.8173

*Note:* Bold values indicate statistically significant relationships (*p* < 0.05).

^a^ First order frequency term.

^b^ Second order frequency term.

Likewise, the relationship between SCBD and the frequency of cyanobacterial species was better explained by a second‐order polynomial relationship, and the predictive power was also high (approximately 72%) (Table 3). However, in this case, there was a quadratic relationship with the SCBD, increasing to a frequency of approximately 40%, and then decreasing (Figure 6). The largest contribution was from *Pseudanabaena*, which presented the highest SCBD value recorded among all groups of algae. On the other hand, phytoflagellates were better described by a positive linear first‐order model, which showed approximately 84% predictive power (Table 4; Figure 6). The species that presented the highest values of contribution to the beta diversity were *Euglena* sp. and *Euglena* sp. 1, both with a frequency of occurrence greater than 50%.

**FIGURE 7:**
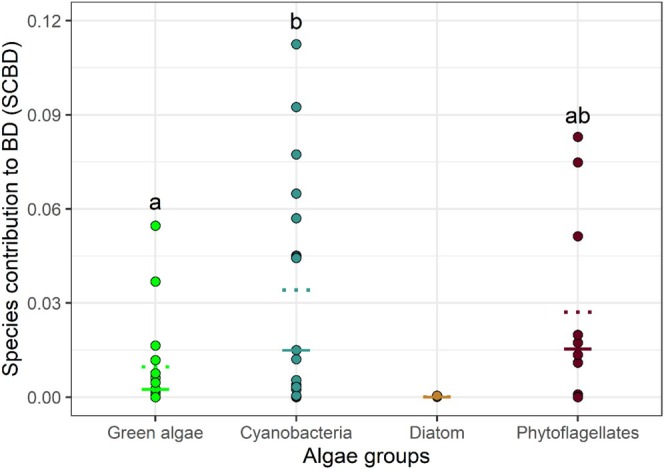
Species contribution to beta diversity (SCBD) in the four major groups. Dashed lines indicate the mean, while solid lines indicate the median for each group. Letters indicate significantly different contributions (*p* < 0.05) according to Tukey's test (a posteriori pairwise comparison).

There was a significant difference (ANOVA *F* = 0.04795, *p* < 0.001) between the SCBD values for the four groups of algae. The Tukey test indicated that the contribution of cyanobacterial species was greater than the contribution of green algae species. The contribution of phytoflagellates was lower and did not differ significantly from cyanobacteria and green algae (Figure 7).

## 5 Discussion

In this study, we partitioned the patterns of beta diversity of the algal community into the contribution of sites (LCBD) and species (SCBD), and we evaluated the factors involved in this variation of community composition. Moreover, species with an intermediate frequency of occurrence influenced the LCBD (quadratic model). Species diversity Shannon‐Weaver Diversity Index (*H*′) and evenness negatively influenced the contribution of each site to beta diversity, indicating that low diversity was influenced by high variation in species abundance between sites. Light availability (% canopy cover and turbidity) negatively affected LCBD. These results showed that the communities in the micro ecosystems were limited by the availability of light, even in those located in the open Restinga, regardless of the size of the habitat (water volume in the bromeliad). The water temperature and oxygen concentration did not influence the LCBD values, probably due to the low variability of these variables in the micro‐ecosystems. The species with accessory frequency were the ones that most contributed to SCBD. Thus, our hypotheses were partially accepted.

### 5.1 Local Contribution to Beta Diversity (LCBD)

There was no relationship between LCBD and species richness. This fact may be directly linked to high values of rare species (73.3%), since there is low variation in species frequency between locations (Pozzobom et al. 2020). High contribution of rare species is common in nature (Siqueira et al. 2012), especially for algae, which are organisms passively dispersed by different vectors, such as wind (Kristiansen 1996) and manage to reach different environments, but nevertheless cannot develop large populations because they may be limited by local conditions. These limiting conditions can be evident in extreme environments with high turbidity (Padisák and Naselli‐Flores 2021), or, as in the case of micro‐ecosystems formed by tank bromeliads with lower water volume.

On the other hand, the results showed a negative relationship between Shannon and Equitability indices with the local contribution to beta diversity. This result suggests that these two diversity indices can provide valuable information about the degree of conservation of environments. High LCBD values indicate environments with specific conditions (Legendre and De Cáceres 2013). The negative relationship of LCBD with the Shannon index indicates that less diverse locations may represent unique locations, and LCBD values can easily predict these environments, making them priorities for management and recovery practices. Since the Shannon index takes into account the relative abundance of species (Shannon and Weaver 1963), it may be more sensitive for detecting changes between local communities other than mere changes in species richness, and is, therefore, a more sensitive and important tool for biomonitoring.

Likewise, evenness provides valuable information, directly reflecting whether the community is governed by many species with uniformly distributed abundances or by a few dominant species (Pielou 1966). In this case, the negative relationship of this index with the LCBD indicates that the microcosms with high Equitability are the ones that differ the least from the other locations. This information can be useful, for example, to detect impacted sites, which often experience declines in species numbers, and only resistant species dominate.

The results showed that, as expected, the variations in algal communities in the micro ecosystems studied were mainly influenced by the availability of light (% canopy cover and turbidity). This result was evidenced by the negative relationships of these two variables with the contribution of microcosms to beta diversity. In the micro ecosystems used, this influence can be even greater, since the entire environment is directly affected by the canopy, while in a lake, for example, exposure to sunlight varies according to location and time of day. In addition, locations with high canopy cover are more susceptible to the entry of organic matter and production of autochthonous material (Farjalla et al. 2016; Céréghino et al. 2020), which increases water turbidity, making these two variables determinants of beta diversity of photosynthetic organisms. Unlike in the species‐area theory (MacArthur and Wilson 1967), the volume of the bromeliads (habitat size) was not the determining factor for the variation in the local contribution to the beta diversity of algae.

### 5.2 Contribution of Species to Beta Diversity (SCBD)

The morphophysiological characteristics of the species, as well as the role they play in the environment, may be associated with SCBD values, since generalist species contribute less to SCBD than species with narrower niches, given that the former have a wider distribution in the environment (Heino 2013a; Yang et al. 2018). High SCBD values may indicate high variation in species abundance, or even influence the differentiation of composition between local communities (Heino 2013b). This may explain the greater importance of cyanobacteria for SCBD. This group, which presented the highest richness and abundance among the analysed groups, showed a quadratic relationship between SCBD values and frequency of occurrence, that is, the importance of taxa increased up to a threshold and then started to fall. This is because when species are very frequent, they start to contribute less to compositional variation (Pozzobom et al. 2020). This was the case in *Cyanodictyon* sp., *Merismopedia tenuissima* Lemmermann and *Pseudanabaena* sp., which reached a frequency of occurrence greater than 50%.

Phytoflagellates presented the second largest contribution to SCBD. Differently from what was found for cyanobacteria, there was a positive linear relationship with the frequency of the species. Thus, despite being very frequent, the density variation of these species contributes to increasing beta diversity, even between environments that share the presence of the same species (Landeiro et al. 2018). Although low light availability (high canopy cover and/or high turbidity) does not affect phytoflagellates intensely, as they are potential mixotrophs (Flynn et al. 2013; Poniewozik et al. 2020), they were positively related to tank volume (habitat size) and water temperature, causing a variation in the abundance of species and, consequently, an increase in SCBD.

### 5.3 Patterns of Algal Diversity in Micro Ecosystems

The variation of communities in natural micro ecosystems was influenced by light availability (% canopy cover) and tank volume (size). Cyanobacteria showed a greater advantage compared to diatoms, probably due to the low amount of silica (i.e., essential nutrient for the formation of the siliceous shell of diatoms) in micro‐ecosystems (de Lyra 1971). In addition, the low contribution of diatoms can be attributed to the high concentration of organic matter and nutrients, especially in micro‐ecosystems with greater canopy coverage (Poniewozik et al. 2020), which may favour groups with greater affinity for nutrients, such as cyanobacteria (Reynolds 2006).

Our findings align with and expand upon previous research, such as Brouard et al. (2011), which identified algae as vital components of tank bromeliad ecosystems, with their biomass strongly influenced by light availability. Our study further refines this understanding by demonstrating that variations in the diversity of algae communities within these ecosystems are shaped by factors regulating light availability, such as water turbidity and canopy cover. This highlights light availability as a critical resource in tank bromeliad ecosystems, influencing the community assembly of various algae and microfauna groups (Busse et al. 2019). Given the role of these groups in driving food web dynamics (Brouard et al. 2011; Farjalla et al. 2016), it is expected that light availability triggers significant bottom‐up effects within the food web of this micro‐ecosystem. This represents an intriguing area for future research, with potential implications for understanding the cascading impacts of environmental factors on ecosystem functioning. Tank‐bromeliad ecosystems are characterised by hosting diverse communities of organisms, ranging from microorganisms to small aquatic invertebrates (Antiqueira et al. 2022), making them ideal for studying beta diversity and its drivers (Antiqueira, Petchey, dos Santos, et al. 2018; Busse et al. 2018). Our study emphasises the ecological uniqueness of sites and species regarding their contributions to beta diversity, highlighting the negative effects of Shannon diversity, turbidity and canopy cover on local contributions to beta diversity (LCBD). Such findings align with other research conducted in bromeliad tanks, which often reveal that these ecosystems harbour distinct compositions of organisms influenced by microenvironmental factors, including light availability, nutrient inputs, temperature and water retention capacity (Antiqueira, Petchey, dos Santos, et al. 2018; Busse et al. 2018; Ramos and Moura 2019; Srivastava et al. 2020).

The high contribution of cyanobacteria and green algae to species diversity in micro‐ecosystems is possible due to their reduced size and small habitat requirements, spending less energy on vital processes, making them more resistant to environmental variations than large‐sized organisms (Willmer 2009; Amundrud et al. 2015). Even larger green algae, such as the filamentous *Oedogonium*, are well adapted to environments with a high variation of dissolved oxygen, being tolerant of the low concentrations present in these microhabitats (Ramos et al. 2018). Environments with acidic pH can favour the occurrence of green algae, as shown by (Lopez et al. 2009). Euglenophytes are also favoured under conditions of high concentration of decomposing organic matter (debris) in the environment (Ramos et al. 2017; Poniewozik et al. 2020; Sachertt Mendes et al. 2020) and especially in shallow environments (Reynolds 2006).

Leaf morphology and the microclimate established in these habitats can directly influence the community that will establish itself in these locations. Bromeliads with greater exposure to light radiation have high water temperature and nutrient concentration (Guimaraes‐Souza et al. 2006; Ramos et al. 2017). Central tanks with wider and shallower wells are more favourable to receiving algal inoculum through air, rain, or even pollinating animals, while narrower and deeper tanks make it difficult for these inoculums to enter (Poniewozik et al. 2020), acting as an environmental filter.

## 6 Conclusion

Our study highlights the critical role of environmental factors, particularly light availability, in shaping beta diversity and structuring algal communities in natural freshwater micro‐ecosystems. Additionally, our findings underscore the value of Local Contributions to Beta Diversity (LCBD) as a key metric for identifying ecologically unique sites that warrant conservation attention. Preserving these distinct microhabitats is essential for maintaining biodiversity at broader spatial scales. From a conservation perspective, our results suggest that habitat heterogeneity, especially variations in light exposure, should be integrated into strategies for protecting freshwater biodiversity. Further research should investigate how environmental variability influences beta diversity in micro‐ecosystems and how these effects scale up to inform conservation strategies at larger spatial scales.

Overall, our study reinforces the significance of tank bromeliads as natural laboratories for ecological research and biodiversity conservation. These microhabitats serve as valuable model systems for studying fundamental ecological processes, including species interactions, nutrient cycling and responses to environmental change (Farjalla et al. 2016; Antiqueira, Petchey, and Romero 2018; Ramos and Moura 2019; Srivastava et al. 2020). Given their capacity to support diverse microbial and algal communities, bromeliad tanks function as important biodiversity reservoirs, particularly in the hyperdiverse Neotropics, where conservation efforts are increasingly urgent. By identifying key environmental drivers of community composition and pinpointing ecologically distinct sites, our findings contribute to advancing ecological theory and guiding conservation initiatives aimed at safeguarding freshwater biodiversity in tropical ecosystems.

## Author Contributions

P.A.P.A., A.B., J.S.P. and G.Q.R. developed the research project and coordinated the field sampling. Y.R.S., B.M.C. and F.M.L. conceived the ideas within this paper. Y.R.S., L.C.R. and P.A.P.A. gathered and organised information with extensive suggestions from Y.R.S. B.M.C. and F.M.L. designed and performed all analyses, while visual results were produced by Y.R.S., B.M.C., F.M.L. and P.A.P.A. wrote the first draft, which was thoroughly reviewed by L.C.R. and P.A.P.A. All authors approved the final version.

## Conflicts of Interest

The authors declare no conflicts of interest.

## Supporting information


**Data S1:** Supporting Information.

## Data Availability

The data that support the findings of this study are available in the Supporting Information of this article.
