# Supplementary material for: Determinants of Cyanobacteria and Algae Diversity in Natural Freshwater Micro‐Ecosystems
Source: Environ Microbiol. 2025 Jul 29;27(8):e70157. doi: 10.1111/1462-2920.70157 (PMC12306149; doi:10.1111/1462-2920.70157)
Supplement: Supplementary file 1 — Data S1: Supporting Information. [file EMI-27-e70157-s001.docx]

**S1**: List of phytoplankton taxa occurring in tank bromeliads. Small (S), medium (M) and large (L) sizes.

| **Taxa** | **Occurrence** | **Frequency [%]** | |
| --- | --- | --- | --- |
| **Green Algae** |  |  |  |
| **Chlorophyceae** | |  |  |
| *Ankyra* sp*.* | M | 1.3 | **Rare** |
| Chlorophyceae not id 1 | L | 1.3 | **Rare** |
| Chlorophyceae not id 2 | S | 2.6 | **Rare** |
| Chlorophyceae not id 3 | M | 1.3 | **Rare** |
| *Monoraphidium convolutum* (Corda) Komárková-Legnerová | S M L | 16.88 | **Rare** |
| *Monoraphidium minutum* (Nägeli) Komárková-Legnerová | M | 1.3 | **Rare** |
| *Monoraphidium* sp. | S M L | 3.9 | **Rare** |
| *Schroederia* sp. | M | 1.3 | **Rare** |
| **Oedogoniophyceae** |  |  |  |
| *Oedogonium reinschii* J.Roy ex Hirn | S M L | 27.27 | **Accessory** |
| *Oedogonium* sp*.* | M L | 5.19 | **Rare** |
| **Trebouxiophyceae** |  |  |  |
| *Crucigenia* sp. | S M L | 22.08 | **Rare** |
| *Oocystis borgei* J.W.Snow | M | 2.6 | **Rare** |
| *Oocystis* sp. | S M L | 16.88 | **Rare** |
| *Oocystis* sp1 | S M L | 41.56 | **Accessory** |
| *Oocystis* sp2 | S M L | 15.58 | **Rare** |
| **Cyanobacteria** |  |  |  |
| *Chroococcus* sp. | S L | 3.9 | **Rare** |
| Cyanobacteria not id1 | L | 1.3 | **Rare** |
| Pseudanabaenaceae not id 1 | L | 1.3 | **Rare** |
| Pseudanabaenaceae not id 2 | M | 1.3 | **Rare** |
| *Cyanodictyon* sp. | S M L | 54.55 | **Constant** |
| *Cyanodictyon* sp1 | S L | 2.6 | **Rare** |
| *Merismopedia tenuissima* Lemmermann | S M L | 58.44 | **Constant** |
| *Merismopedia* sp. | S M L | 36.36 | **Accessory** |
| *Merismopedia* sp1 | S M L | 12.99 | **Rare** |
| *Oscillatoria* sp. | M | 1.3 | **Rare** |
| *Planktolyngbya* sp. | S M | 6.49 | **Rare** |
| *Planktolyngbya* sp1 | L | 2.6 | **Rare** |
| *Pseudanabaena* sp. | S M L | 57.14 | **Constant** |
| *Romeria* sp. | S M L | 28.57 | **Accessory** |
| *Synechococcus* sp. | S M L | 15.58 | **Rare** |
| *Synechocystis* sp. | S M L | 31.17 | **Accessory** |
| *Synechocystis* sp1 | S M L | 38.96 | **Accessory** |
| **Diatoms** |  |  |  |
| **Bacillariophyceae** |  |  |  |
| *Achnantes* sp. | M | 1.3 | **Rare** |
| **Coscinodiscophyceae** |  |  |  |
| *Aulacoseira granulata* (Ehr.) Sim. var*. granulata* | S | 1.3 | **Rare** |
| **Mediophyceae** |  |  |  |
| *Cyclotella* sp. | S | 1.3 | **Rare** |
| **Phytoflagellates** |  |  |  |
| **Chlamydophyceae** |  |  |  |
| *Chlamydomonas* sp. | S L | 6.49 | **Rare** |
| **Cryptophyceae** |  |  |  |
| *Cryptomonas* sp. | S M L | 11.69 | **Rare** |
| Cryptophyceae not id 1 | S M L | 22.08 | **Rare** |
| **Euglenophyceae** |  |  |  |
| *Euglena* sp. | S M L | 54.55 | **Constant** |
| *Euglena* sp1 | S M L | 55.84 | **Constant** |
| *Euglena* sp2 | S M L | 32.47 | **Accessory** |
| *Euglena* sp3 | S M L | 18.18 | **Rare** |
| *Euglena* sp4 | M | 1.3 | **Rare** |
| *Phacus* sp. | L | 1.3 | **Rare** |
| *Trachelomonas* sp. | S M L | 1.3 | **Rare** |
